# The risk of sexual transmission of HIV in individuals with low-level HIV viraemia: a systematic review

**DOI:** 10.1016/S0140-6736(23)00877-2

**Published:** 2023-08-05

**Authors:** Laura N Broyles, Robert Luo, Debi Boeras, Lara Vojnov

**Affiliations:** a Global Health Impact Group, Atlanta, GA, USA; b WHO, Geneva, Switzerland

## Abstract

**Background:**

The risk of sexual transmission of HIV from individuals with low-level HIV viraemia receiving antiretroviral therapy (ART) has important public health implications, especially in resource-limited settings that use alternatives to plasma-based viral load testing. This Article summarises the evidence related to sexual transmission of HIV at varying HIV viral load levels to inform messaging for people living with HIV, their partners, their health-care providers, and the wider public.

**Methods:**

We conducted a systematic review and searched PubMed, MEDLINE, Cochrane Central Register of Controlled Trials, Embase, Conference Proceedings Citation Index-Science, and WHO Global Index Medicus, for work published from Jan 1, 2010 to Nov 17, 2022. Studies were included if they pertained to sexual transmission between serodiscordant couples at various levels of viraemia, the science behind undetectable=untransmittable, or the public health impact of low-level viraemia. Studies were excluded if they did not specify viral load thresholds or a definition for low-level viraemia or did not provide quantitative viral load information for transmission outcomes. Reviews, non-research letters, commentaries, and editorials were excluded. Risk of bias was evaluated using the ROBINS-I framework. Data were extracted and summarised with a focus on HIV sexual transmission at varying HIV viral loads.

**Findings:**

244 studies were identified and eight were included in the analysis, comprising 7762 serodiscordant couples across 25 countries. The certainty of evidence was moderate; the risk of bias was low. Three studies showed no HIV transmission when the partner living with HIV had a viral load less than 200 copies per mL. Across the remaining four prospective studies, there were 323 transmission events; none were in patients considered stably suppressed on ART. Among all studies there were two cases of transmission when the index patient's (ie, patient with previously diagnosed HIV infection) most recent viral load was less than 1000 copies per mL. However, interpretation of both cases was complicated by long intervals (ie, 50 days and 53 days) between the transmission date and the most recent index viral load result.

**Interpretation:**

There is almost zero risk of sexual transmission of HIV with viral loads of less than 1000 copies per mL. These data provide a powerful opportunity to destigmatise HIV and promote adherence to ART through dissemination of this positive public health message. These findings can also promote access to viral load testing in resource-limited settings for all people living with HIV by facilitating uptake of alternative sample types and technologies.

**Funding:**

Bill & Melinda Gates Foundation.

## Introduction

Viral load testing is the gold standard for monitoring the response to HIV antiretroviral therapy (ART) with the goal of durable suppression of viraemia to both promote health and longevity and decrease the risk of transmission. As access to ART and viral load monitoring has increased, data from various settings show that a small minority of people living with HIV on ART have viral loads that are detectable but below the threshold for virological failure (ie, 1000 copies per mL).^1^, ^2^, ^3^ The clinical significance and management of this low-level viraemia has been an ongoing topic of debate. At the individual level, low-level viraemia has been associated with virological failure, HIV drug resistance, and worse clinical outcomes; however, data on these outcomes in patients taking integrase inhibitors are scarce.^4^, ^5^

From a public health perspective, low-level viraemia can also have implications in disease transmission risks and thus affect messaging for people living with HIV, including undetectable=untransmittable (U=U) campaigns.^6^ Although it is generally accepted that HIV viral loads of less than 200 copies per mL are associated with zero risk of sexual transmission and this threshold is used for U=U messaging in many high-income settings,^7^ the risk at virus levels higher than 200 copies per mL has been controversial. This issue is of particular concern in resource-limited settings where alternative viral load testing methods (eg, dried blood spots and point-of-care platforms) are widely used because plasma-based testing on centralised molecular platforms is not feasible.^8^, ^9^ These innovative sample types and technologies have enabled a rapid and substantial increase in access to viral load monitoring in low-income and middle-income countries. However, these alternative approaches to viral load quantification have variable diagnostic accuracy when low virological thresholds are used.^10^

**Research in context**

**Evidence before this study**

Low-level viraemia in people living with HIV on antiretroviral therapy (ART) has implications for both individual health outcomes (eg, virological failure and HIV drug resistance) and public health messaging around transmission risks. Prevention campaigns such as undetectable=untransmittable provide positive messages that promote treatment adherence and decrease HIV stigma. It has generally been widely accepted that viral loads less than 200 copies per mL have no risk of sexual HIV transmission; however, the risk of transmission at higher ranges of low-level viraemia (ie, 200–999 copies per mL) is more controversial. This is of particular relevance in resource-limited settings that use alternative viral load technologies, such as dried blood spots, to expand access to testing. The limitations of non-plasma-based viral load testing technologies have raised questions about the risks of sexual HIV transmission with low-level viraemia and the implications for public health messaging.

**Added value of this study**

We searched PubMed, MEDLINE, Cochrane Central Register of Controlled Trials, Embase, Conference Proceedings Citation Index-Science, and WHO Global Index Medicus databases, as well as relevant conference proceedings, for work published from Jan 1, 2010 to Nov 17, 2022, using broad search terms (“HIV”, “viral load”, “low-level viremia”, and “transmission”). Input from content experts on additional relevant cases and studies was also solicited. Eight studies were identified related to sexual transmission of HIV at varying HIV viral loads. In more than 7700 serodiscordant couples, only two potential transmissions were found when the index partner's viral load was less than 1000 copies per mL. In both cases, viral load testing of the index partner was conducted at least 50 days from the transmission event, complicating interpretation. This study is the first to collate evidence on sexual transmission of HIV at low levels of viraemia and address the risks with viral loads of 200–1000 copies per mL.

**Implications of all the available evidence**

The findings of this systematic review are important for ensuring dissemination of clear, consistent, accurate, and positive messages about the almost zero risk of sexual transmission of HIV in individuals with low-level HIV viraemia. Although an undetectable viral load is the goal for all people living with HIV on ART, these data demonstrate that the risk of sexual transmission of HIV at low-level viraemia is almost zero. These results should enable development and widespread dissemination of impactful prevention campaigns for all settings, including those without consistent access to traditional plasma-based viral load testing. Instilling confidence in all viral load results, coupled with positive messaging, is essential to expand access to treatment monitoring, promote better quality of care for all people living with HIV on ART, and reduce stigma and discrimination.

The public health community is actively seeking to address the transmission implications of low-level viraemia and ensure that the most accurate and ethical messaging is provided to people living with HIV, their sexual partners, their health-care providers, and the public. To help to clarify this crucial issue, this Article summarises the evidence related to sexual transmission of HIV at varying levels of HIV viral load with a focus on determining the risk of sexual transmission of HIV at viral loads less than 1000 copies per mL.

## Methods

### Study design

The systematic review described in this Article was one component of a larger systematic review conducted on Sept 15, 2020, to determine whether the HIV treatment failure threshold should be reduced from 1000 copies per mL.^11^ The original systematic review covered four outcomes: virological failure, disease progression, drug resistance, and HIV transmission. The current review focuses specifically on the findings related to sexual HIV transmission; it does not include transmission from parent to child.

### Search strategy and selection criteria

The systematic review was conducted according to PRISMA,^12^ following a predefined study protocol. The full PRISMA checklist is available in the appendix. A search strategy was prepared to extract all potentially relevant studies. We searched PubMed, MEDLINE, Cochrane Central Register of Controlled Trials, Embase, Conference Proceedings Citation Index-Science, and WHO Global Index Medicus for literature published from Jan 1, 2010 to June 30, 2020 without restrictions on language, age, geography, document type, or publication status, following international guidelines. Studies before Jan 1, 2010, were not initially considered due to the use of older, less precise viral load technologies^13^ and the desire to focus on the period after viral load testing became recommended by WHO with specific thresholds for treatment failure.

We also searched conference abstracts using the Conference on Retroviruses and Opportunistic Infections, International Conference on AIDS and Sexually Transmitted Infections in Africa, African Society for Laboratory Medicine, and International AIDS Society conference websites. Bibliographies of screened and selected studies, as well as review articles, were also reviewed. Content experts identified key studies from before Jan 1, 2010 for inclusion and provided further data when relevant.

In searching each database and conference website, we used the terms “(HIV) AND (Viral Load OR Virus Load* OR Viral Burden* OR Virus Burden* OR Virus Titer* OR Viral Titer* OR Virus Titre* OR Viral Titre* OR VL OR VLs) AND (“low level” OR “low level viremia” OR “viral load threshold”) AND (transmission or HIV infection)”.

All relevant studies were independently reviewed in full by two authors (RL and DB). Studies for the HIV transmission outcome were included if they met the following criteria: pertained to sexual transmission between serodiscordant couples at various levels of viraemia, pertained to the science behind U=U, and evaluated the public health effect of low-level viraemia. Studies were excluded if they did not specify viral load thresholds or a definition for low-level viraemia or did not provide quantitative viral load information for relevant transmission outcomes. Reviews, non-research letters, commentaries, and editorials were also excluded.

On Nov 17, 2022, an identical, updated search was repeated with a focus on outcomes related to sexual HIV transmission to include articles published between July 1, 2020 and Nov 17, 2022. Content experts were also contacted to identify other studies that might contain relevant information with no restrictions on publication date. A third reviewer (LV) was available in case of discrepancies or disagreements in study selection; however, none were encountered.

### Study certainty and bias assessment

Study certainty (eg, inconsistency, indirectness, and imprecision) was evaluated using a tool created based on Grading of Recommendations, Assessment, Development and Evaluation; Quality Assessment of Diagnostic Accuracy Studies 2; and Standards for Reporting of Diagnostic Accuracy Studies criteria.^14^, ^15^, ^16^ Potential risk of bias for each study was assessed independently by two authors (LNB and RL) using the ROBINS-I framework.^17^ Certainty and risk of bias was assessed for each study and, based on the results of each study, an overall certainty and risk of bias was determined for the entire body of evidence.

### Data analysis

Data from each of the studies were extracted and summarised by two authors (LNB and RL) outlining their principal components, including sample size, study design, patient characteristics, thresholds examined, and relevant study outcomes. Data from all studies were summarised descriptively. When quantitative data were available and common across comparable studies, such as sample sizes and proportions, frequency statistics were used to describe the populations evaluated and their results.

### Role of the funding source

The funder of the study had no role in study design, data collection, data analysis, data interpretation, or writing of the report.

## Results

The initial search for the comprehensive review identified 241 potential studies for evaluation, of which 31 were included in the original review. Of those, seven focused on HIV transmission;^18^, ^19^, ^20^, ^21^, ^22^ two were related to vertical (ie, parent to child) transmission and, thus, not included in this analysis.^23^, ^24^ No identified studies evaluated the transmissibility of HIV through the sharing of injection drug use equipment when a person's viral load is less than 1000 copies per mL. 174 studies that did not include intervention and a comparator were excluded for not evaluating the effect of low-level viraemia or a viral load threshold, and 36 studies were excluded for not meeting the other study criteria (figure).

**Figure:**
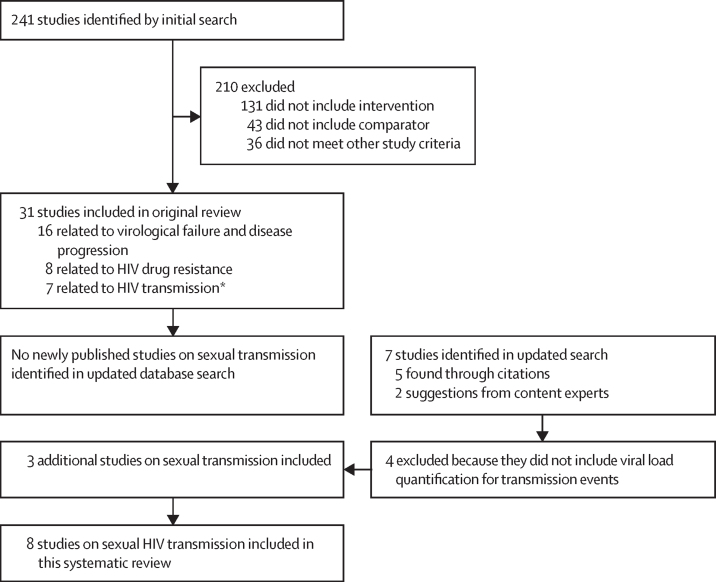
Study selection *Five sexual transmissions and two parent-to-child transmissions.

The updated and expanded search on Nov 17, 2022 found no studies published since the original search that included sexual transmission outcomes at varying HIV viral loads. Content experts and citation review identified three additional papers—two published before Jan 1, 2010, and one published in 2016—that provided information relevant to sexual transmission at low levels of viraemia. A total of eight papers focused on sexual transmission of HIV at low levels of viraemia were included in this systematic review (table 1).^26^, ^27^, ^28^

**Table 1:** Characteristics of included studies

	**Study name**	**Countries**	**Study type**	**Study years**	**Type of serodiscordant couples**	**Number of couples**	**Number of linked transmissions during study**
Bavinton et al (2018)^18^	Opposites Attract	Australia, Brazil, and Thailand	Cohort	2012–16	100% male–male couples	358	0
Cohen et al (2016)^19^	HPTN 052	Nine countries worldwide	RCT	2005–10	97% male–female couples and 3% unspecified	1763	46
Fideli et al (2001)^25^	..	Zambia	Nested case-control within prospective cohort	1994–2000	100% male–female couples	1022 (case-control: 109 index transmitters and 208 non-transmitting controls)	129
Mujugira et al (2016)^26^	Partners PrEP Study	Kenya and Uganda	Analysis of couples in placebo arm of RCT	2008–12	100% male–female couples	1573	Index not on ART: 55; index on ART: 3
Quinn et al (2000)^20^	Rakai	Uganda	RCT	1994–98	100% male–female couples	415	90*
Rodger et al (2016)^21^	PARTNER	14 European countries	Cohort	2010–14	62% male–female couples and 38% male–male couples	1166	None
Rodger et al (2019)^22^	PARTNER2	14 European countries	Cohort	2010–17	100% male–male couples	972	None
Tovanabutra et al (2002)^27^	..	Thailand	Cross-sectional study of wives of HIV-positive men	1992–98	100% male–female couples	493	44% of female partners found to have an HIV-positive serostatus at enrolment*

ART=antiretroviral therapy. RCT=randomised controlled trial.

* Phylogenetic linkage analysis not conducted.

Following the GRADE framework, certainty was assessed with regards to potential inconsistency, indirectness, and imprecision of results. There was determined to be no serious risk of inconsistency or imprecision since several large, well characterised randomised controlled trials and cohort studies were available for review. However, there was serious indirectness because suboptimal data were available to assess the viraemic episodes (ie, the period in which the patient's viral load was detectable) associated with transmission. This indirectness created uncertainty about when the HIV transmission event occurred and the viral load level at the time of transmission. On the basis of these assessments, the overall certainty of the evidence was rated as moderate.

The risk of bias findings for each study is outlined in table 2. Although one paper had some overall risk of bias based on its cross-sectional nature, the overall risk of bias was considered low, as the remaining studies yielded no major concerns about how patients were enrolled or how outcomes were measured and reported.

**Table 2:** Risk of bias

	**Randomised trials: bias from randomisation**	**Non-randomised trials: bias from confounding**	**Non-randomised trials: bias from participant selection**	**Non-randomised trials: bias in intervention classification**	**All: bias from deviation from intended intervention**	**All: bias from missing outcome data**	**All: bias in outcome measurement**	**All: bias in selection of reported results**	**Overall risk of bias judgment**
Bavinton et al (2018)^18^	NA	Low	Low	Low	Low	Low	Low	Low	Low
Cohen et al (2016)^19^	Low	NA	NA	NA	Low	Low	Low	Low	Low
Fideli et al (2001)^25^	NA	Low	Some	Low	Low	Low	Low	Low	Low
Mujugira et al (2016)^26^	Low	NA	NA	NA	Low	Low	Low	Low	Low
Quinn et al (2000)^20^	Low	NA	NA	NA	Low	Low	Low	Low	Low
Rodger et al (2016)^21^	NA	Low	Low	Low	Low	Low	Low	Low	Low
Rodger et al (2019)^22^	NA	Low	Low	Low	Low	Low	Low	Low	Low
Tovanabutra et al (2002)^27^	NA	Some	Some	Some	Low	Low	Low	Low	Some

NA=not applicable.

Eight studies on sexual HIV transmission were included in the systematic review, consisting of four cohort studies, three randomised controlled trials, and one cross-sectional study across 25 different countries (table 2). The analyses included 7762 serodiscordant couples, most of which were male–female couples. The cohort studies enrolled serodiscordant couples to evaluate for sexual transmission of HIV. These randomised controlled trials assigned participants to either ART or HIV prevention-based interventions and followed up participants in both the intervention and control groups for HIV transmission events between study participants and their partners.

Three of the studies (Opposites Attract,^18^ PARTNER,^21^ and PARTNER2^22^) showed no evidence of HIV transmission between the serodiscordant couples when the partner living with HIV had a viral load of less than 200 copies per mL. Notably, these studies either excluded couples with a viral load greater than 200 copies per mL (ie, PARTNER and PARTNER2) or had very few instances of participants with a viral load greater than 200 copies per mL and no linked transmission (ie, Opposites Attract).

Among the three randomised controlled trials there were multiple transmission events that allowed an examination of associated viral loads. Quinn and colleagues' Rakai study^20^ described 415 male–female serodiscordant couples in Uganda that were retrospectively identified from within a large community-based trial conducted in 1994–98. During the study, participants were followed up for a median of 22 months; no participants received ART. In the 90 couples in which the partner seroconverted, index partner viral loads were retrospectively analysed, and the viral load timing was an average of 4 months before seroconversion. Phylogenetic testing to confirm linkage was not conducted. Analysis showed that there were no instances of transmission in the 51 couples in which the HIV-positive partner had a viral load of less than 1500 copies per mL. Among the 90 seroconversions, only five (6%) of those occurred with an index partner (ie, patients with a previously diagnosed HIV infection) with a viral load of 1500–3499 copies per mL.

The HPTN 052 study^19^ examined 1763 serodiscordant couples in which the partner living with HIV was randomly assigned to either early ART (defined as CD4 count of 350–550 cells per μL) or delayed ART (defined as two consecutive CD4 counts of less than 250 cells per μL or the development of an AIDS-defining illness). The study endpoint was genetically linked HIV infection in the previously HIV-negative partner. In participants who initiated ART, 72% received zidovudine, lamivudine, and efavirenz and were followed up with viral load measurements every 3 months. ART treatment failure was defined as two viral loads greater than 1000 copies per mL on two consecutive visits after initial suppression. Treatment failure occurred in 45 (5%) of the 886 participants in the early-ART group and five (3%) of the 184 participants in the delayed-ART group. During the study, there were 46 linked infections—three in the early ART group and 43 in the delayed ART group. No linked infections were observed when HIV infection was stably suppressed by ART (less than 1000 copies per mL) in the index participant.

Eight of the linked partner infections occurred after the index partner started ART. In four cases, the partner was diagnosed with HIV infection less than 90 days after the index patient started ART. In the remaining four cases, infection occurred after treatment failure. Of the four cases that occurred after treatment failure, there was a single linked transmission event in which the index viral load was less than 1000 copies per mL. In that case, the index patient was receiving second-line ART after treatment failure on the initial regimen. The estimated partner seroconversion occurred 4·5 years after ART initiation and the partner with HIV's most recent viral load measurement before transmission was 617 copies per mL. This viral load result was drawn 50 days before the estimated infection date.

The Partners PrEP (pre-exposure prophylaxis) Study was a randomised controlled trial of 4747 heterosexual serodiscordant couples in Kenya and Uganda conducted in 2008–12.^26^ A subanalysis of 1573 HIV serodiscordant couples enrolled in the placebo group of the Partners PrEP Study who were followed up for 2979 person-years found that no linked transmission events occurred in couples in which the index partner was on ART for more than 6 months. There were three genetically linked transmissions in which the index partner self-reported ART use; in all three couples, seroconversion occurred within 6 months of ART initiation (duration of ART range 0–149 days). Because the participants' blood was drawn every 6 months relative to the date of enrolment (not date of ART initiation), there is substantial uncertainty around the index patient's viral load at the time of transmission for each couple. Notably, one index patient had pre-ART viral load results of 694 copies per mL at enrolment and 824 copies per mL before ART initiation. The post-ART viral load—taken 86 days after initiation—was 872 copies per mL, suggesting suboptimal ART regimen adherence even when taking the low HIV setpoint (ie, an individual's steady state viral load during chronic HIV infection) into consideration. Transmission occurred between this patient and her partner approximately 149 days after ART initiation (ie, 53 days after viral load quantification). This transmission could have occurred when the viral load was less than 1000 copies per mL; however, the potential absence of ART exposure and the 53-day gap between seroconversion and time of viral load quantification makes it difficult to determine the true dynamics of this event.

In a cohort study conducted by Fideli and colleagues^25^ from 1994 to 2000 in Zambia, 1022 serodiscordant couples were followed up for a median of 15 months. Viral load testing was conducted every 3 months and no participants received ART. 129 linked transmissions occurred over the course of the study. A nested case-control study comparing 109 people in which HIV was documented to be transmitted from one partner to the other with 208 controls in which no transmission of HIV was documented found no transmissions when the index partner had a viral load of less than 1000 copies per mL and 96 (92%) of 104 index partners who transmitted had a viral load of greater than 10 000 copies per mL.

Finally, in Thailand in 1992–98, Tovanabutra and colleagues^27^ conducted a cross-sectional study of 493 heterosexual serodiscordant couples in which male blood donors found to be positive for HIV at blood donation were invited to bring in their wives for evaluation. Couples were enrolled if, with the exception of sexual activity with their husband, the wives had no HIV risk behaviours and both partners agreed to HIV testing and an interview on behaviour and demographic factors. Viral load testing was done at the time of enrolment and no participants were already on ART. At enrolment, 218 (44%) of the wives were found to be living with HIV; phylogenetic analysis was not done to confirm linkage. Results showed that no transmission had occurred in women whose husbands had baseline viral loads of less than 1094 copies per mL.

## Discussion

This systematic review of sexual transmission of HIV in individuals with low-level HIV viraemia on ART comprised eight studies and more than 7700 serodiscordant couples. Among the documented transmission events, only two were potential transmissions when the index partner's viral load was less than 1000 copies per mL. However, in both cases the viral load test was done at least 50 days before the transmission occurrence. This fact, coupled with the inherent variability in molecular viral load results,^13^, ^29^ complicates interpretation of the events. In the case in the Partners PrEP Study, the index partner's pre-ART viral load was less than 1000 copies per mL at two timepoints before ART initiation, suggesting a low setpoint. The absence of a clinically significant viral load decrease, even when reportedly on ART, indicates that the index patient might have had challenges with ART adherence. In both cases, no other potential contributing factors (eg, concurrent sexually transmitted infection) were noted. Taken together, this systematic review found no definitive evidence of HIV transmission when viral loads were less than 600 copies per mL and an incredibly rare occurrence of possible transmissions with viral loads between 600 and 1000 copies per mL. Further, studies of infectivity have shown that the estimated per act risk of sexual transmission without a condom when the index partner had a viral load of 1000 copies per mL is extremely small (0·00028), lending credence to these findings.^30^

It is also worth noting that in the studies that provided the full range of viral loads in the index partners, transmissions occurred at viral loads much higher than 1000 copies per mL. Index partners with viral loads greater than 10 000 copies per mL constituted 81% and 92% of transmissions in the Rakai and Zambia studies, respectively. Similarly, in the HPTN 052 transmission events in which the index partner was taking ART, the viral loads of the index partners ranged from 43 486 copies per mL to more than 750 000 copies per mL.

By demonstrating that the risk of sexual transmission of HIV is almost zero when the index partner has a viral load less than 1000 copies per mL, our findings underscore the importance of prevention campaigns, while also suggesting that the U=U message applies to people living with HIV experiencing low-level viraemia. This message is crucial for low-income and middle-income countries where the disease and infrastructure burdens are high and national programmes are often reliant on alternative sample types and technologies to fully expand access of viral load testing to all people living with HIV. Further, it might encourage more positive and clear messaging on the role of ART in preventing transmission to sexual partners. It will be essential, however, to ensure that the potential individual health risks of low-level viraemia (eg, virological failure and drug resistance) are clearly communicated and that providers reinforce the goal of an undetectable viral load for optimal personal health outcomes.

Although the data quality was moderate, limitations of this systematic review included the varied definitions for low-level viraemia used in the studies, along with differences in the timing and frequency of viral load testing and patient follow-up, causing imprecision within the data. Despite these drawbacks, the data summarised in this Article are crucial because the possibility of deriving more definitive evidence on the risk of sexual transmission at low-level viraemia is most likely not feasible given current HIV standards of care (ie, ART for all people living with HIV) and the requirement for a very large sample size given the very low number of likely transmission events. The need for frequent viral load testing to better determine viral load levels at time of transmission would also be logistically challenging and highly resource-intensive.

It is important to emphasise that these messages and results do not apply to transmission from mother to child. Because vertical transmission can occur during pregnancy (ie, in utero), during childbirth, or through breastfeeding, the duration and intensity of exposure to viraemia is considerably higher than that of sexual transmission. There are also differences in transmission dynamics (eg, direct microtransfusions of maternal blood across the placenta to the fetus in utero or during delivery) that are distinct from sexual transmission.^31^ Vertical transmission has been documented with very low-level viraemia in the intrapartum and postpartum timeperiods, and ensuring undetectable viral loads in pregnant and breastfeeding women is essential for averting new paediatric HIV infections.^22^, ^32^ There are also no data on risk of transmission through sharing of injection drug use equipment at varying levels of viraemia.

Low-level viraemia could have important health implications for individual patients; however, these outcomes have not been thoroughly studied in the context of current, optimal integrase inhibitor-based antiretroviral therapy. An undetectable viral load should be the ultimate goal for clinical management of all people living with HIV on ART. However, the evidence showing almost zero risk of sexual transmission when HIV viral loads are less than 1000 copies per mL provides a powerful opportunity to destigmatise people who are living with HIV and promote adherence to ART through dissemination of this positive public health message.

## Data sharing

All data are available in the manuscript.

## Declaration of interests

We declare no competing interests.
